# Volatile Organic Compounds and Physiological Parameters as Markers of Potato (*Solanum tuberosum* L.) Infection with Phytopathogens

**DOI:** 10.3390/molecules27123708

**Published:** 2022-06-09

**Authors:** Aleksandra Steglińska, Katarzyna Pielech-Przybylska, Regina Janas, Mieczysław Grzesik, Sebastian Borowski, Dorota Kręgiel, Beata Gutarowska

**Affiliations:** 1 Department of Environmental Biotechnology, Lodz University of Technology, Wólczańska 171/173, 90-530 Łódź, Poland; sebastian.borowski@p.lodz.pl (S.B.); dorota.kregiel@p.lodz.pl (D.K.); beata.gutarowska@p.lodz.pl (B.G.); 2 Institute of Fermentation Technology and Microbiology, Lodz University of Technology, Wólczańska 171/173, 90-530 Łódź, Poland; katarzyna.pielech-przybylska@p.lodz.pl; 3 The National Institute of Horticultural Research, Konstytucji 3 Maja 1/3, 96-100 Skierniewice, Poland; regina.janas@inhort.pl (R.J.); mieczyslaw.grzesik@inhort.pl (M.G.)

**Keywords:** seed potatoes, phytopathogens, phytopathogen volatiles, potato physiology, markers, soft rot, early blight, dry rot

## Abstract

The feasibility of early disease detection in potato seeds storage monitoring of volatile organic compounds (VOCs) and plant physiological markers was evaluated using 10 fungal and bacterial pathogens of potato in laboratory-scale experiments. Data analysis of HS-SPME-GC-MS revealed 130 compounds released from infected potatoes, including sesquiterpenes, dimethyl disulfide, 1,2,4-trimethylbenzene, 2,6,11-trimethyldodecane, benzothiazole, 3-octanol, and 2-butanol, which may have been associated with the activity of *Fusarium sambucinum*, *Alternaria tenuissima* and *Pectobacterium carotovorum*. In turn, acetic acid was detected in all infected samples. The criteria of selection for volatiles for possible use as incipient disease indicators were discussed in terms of potato physiology. The established physiological markers proved to demonstrate a negative effect of phytopathogens infecting seed potatoes not only on the kinetics of stem and root growth and the development of the entire root system, but also on gas exchange, chlorophyll content in leaves, and yield. The negative effect of phytopathogens on plant growth was dependent on the time of planting after infection. The research also showed different usefulness of VOCs and physiological markers as the indicators of the toxic effect of inoculated phytopathogens at different stages of plant development and their individual organs.

## 1. Introduction

Phytopathogen infection leads to changes in plant physiology. They involve both secondary metabolism based on inducing the defense programs of a plant as well as primary metabolism, which affects the growth and development of the plant [1]. It is known that both microorganisms and plants emit volatile compounds, including odors that are characteristic of a particular species. These compounds are released as an effect of pathogen activity, and therefore can serve as markers of microbial contamination. Potato flavor has been extensively studied due to its importance in nutrition: volatile compounds predominantly include aldehydes, alcohols, ketones, acids, esters, hydrocarbons, amines, furans, and sulfur compounds. However, the pattern and volatility of the components released from potatoes can differ significantly, depending on whether potatoes are raw or cooked. Moreover, potato pathogens: fungi, bacteria, parasitic plants, viruses, nematodes, and protozoa can modify the volatile pattern emitted from pathogenic species. Potatoes inoculated with pathogens produce volatile molecules that can be considered as the markers of infection [2]. Despite the knowledge on potato volatilome, there is a lack of expertise on volatile compounds emitted from pathogen cultures growing onto potatoes. These microbial volatile products, such as alcohols, ketones, terpenoids, esters, and others, can also be used as the markers of microbial contamination. 

Microbial volatile organic compounds (mVOCs) are carbon-based, secondary metabolites of molds and bacteria characterized by low molecular mass, and easy evaporation allowing their easy disperse in the atmosphere. Most of them produce distinctive odors [3]. More than 1800 unique bacterial and fungal VOCs have been so far reported in the literature [4]. Dozens or even hundreds of microbial volatile organic compounds are produced by the same microorganisms, but some of them are related to specific fungal or bacterial metabolism [5]. The composition of VOCs significantly differs between two species of molds and even two isolates belonging to the same species [6]. Except strain specify, volatile production is also influenced by growth medium, incubation time, humidity, and temperature [7]. In addition, for plant VOC, this volatile compound profile is specific for different varieties of the same plant species [8]. 

Some volatile compounds were identified as potential indicators of microbial plant infection. For example, menthol and menthone have been reported as potential markers of açaí pulp infection with the genus *Colletotrichum* [9]. This mold is also responsible for black dot disease of potatoes [10]. In turn, ethanol, ethyl formate, ethyl acetate, and four other mVOCs were indicated as markers of onion infestation by *Fusarium* species [6].

Volatile organic compounds determination in the early stage of pathogen infection was found to be useful in preventing risk of mycotoxin production on wheat [11] and walnut [12] by toxicogenic *Aspergillus*, *Fusarium,* and *Penicillium* species. Josselin and co-workers [13] distinguished crop infection with *A. flavus* strain (aflatoxin B1 producer) from non-toxicogenic strain by mVOCs evaluation. 

Despite the data relating to the markers of different plants, there is a lack of reliable and commonly used markers assessing the physiological activity, seed value, and yield potential of seed potatoes, which would allow to quickly demonstrate the effects of various treatments, predict plant growth in the field, and be an alternative to long-term and costly field tests. These markers are particularly desirable for monitoring the yield potential of seed potatoes treated against disease, pests, and aging. Moreover, identification of the relationship between selected metabolic processes in stored or treated potato tubers and plant development may be useful in forecasting methods for early recognition of the quality of seed potatoes and their yielding potential [14]. The evaluation of the sowing value of stored and treated seed potatoes is mainly limited to field tests based on the measurement of plant height and tuber yield. The published data do not provide information that would indicate independence of the changing parameters of seed potato quality from selected plant physiological functions, which could be proposed as useful indicators of their seeding value, as previously developed for apple, willow, vegetable species, sorghum, and Jerusalem artichoke [15,16,17,18,19,20,21,22]. The proper understanding of the physiology and its optimization requires a precise physiological "calibration" of the behavior of seed potatoes and the use of appropriate markers that would indicate the quality of seed potato [23,24]. Their use in laboratory conditions allows to quickly evaluate the impact of seed potato treatments on the development, health, and physiological activity of stems and roots, changes in soil structure, and forecasting plant growth in the field.

The traditional methods used to identify pathogen species involve the microscopic inspection of morphological characteristics. Taxonomic keys and descriptions have been developed and widely used for identifying potatoes. Moreover, a loop-mediated isothermal amplification (LAMP) method has been implemented for the rapid and accurate detection of pathogens in plants [25,26]. However, a significant constraint in LAMP assays lies in the design of proper primers. In addition, polymerase chain reaction (PCR) has been used as a tool to detect and quantify the causal agents of late blight (*Phytophthora infestans*), pink rot (*Phytophthora erythroseptica*), leak (*Pythium ultimum*), dry rot (*Fusarium sambucinum*), and soft rot (*Pectobacterium carotovorum*) in potato tubers [27,28]. To detect all possible fungal and oomycete pathogens causing pink rot, watery wound rot, and gangrene in potatoes, specific primers, and probes have been designed for PCR assays [29]. 

In our research, we decided to detect volatile organic metabolites produced by potato pathogens by HS-SPME-GC-MS and try to connect these data with potato physiology. The advantage of using GC-MS in the metabolomic evaluation of VOC is related to the reliability, efficiency, reproducibility, and selectivity of the method. It is able to measure hundreds of compounds due to high chromatographic resolution. Rich libraries for GC-MS are another positive aspect of using this method for VOC analysis [30]. Headspace solid-phase microextraction (HS-SPME) is used for complex matrices analysis. It does not destruct the analyzed sample and does not require the use of organic solvents [8].

Previously, gas chromatography–mass spectrometry (GC-MS) has been used for profiling VOCs in order to differentiate potato tubers infected by rot pathogens [31] and to select tomato varieties with resistance to Fusarium wilt disease [32].

Another method for the detection of different types of volatile organic compounds is the employment of an electronic nose [33]. The method of operation of this device, which is increasingly used, is based on mimicking the sense of smell [34]. 

The aim of the study was to investigate the effect of seed potato phytopathogens on the production of volatile organic compounds (VOC) and on selected growth parameters, physiological activity, and yielding of plants, in order to determine their usefulness as markers in the assessment of the development of potatoes subjected to disease protection treatments.

## 2. Results and Discussion

Potato pathogens can lead to significant losses in storage. Infections occur via wounds that result from harvesting in non-optimal storage conditions. Effective control of potato pathogens during storage requires rapid and accurate tools for diagnosis, and earlier diagnostics may result in better management. In this work, we visually assessed the symptoms of potato pathogens infections caused by both bacterial and fungal pathogens. Then, HS-SPME-GC-MS was used to detect volatile organic compounds produced by these pathogens. PCA and hierarchical cluster analysis were performed. Markers in the form of VOC were proposed for general potato infection and for each pathogen. Afterward, the selected growth parameters were determined under greenhouse conditions. 

Visual inspection has been a common method to identify disease symptoms. The visual assessment of the potato infestation with pathogens is summarized in Table 1.

A variety of fungal and bacterial phytopathogens can cause severe potato diseases during storage. *Fusarium* species are the most common fungal pathogens that attack potatoes. They are responsible for dry rot, characterized by wrinkled and sunken brown or black tissue parts as well as cottony white or purple mycelial and spore pads concentrically arranged on the potato tuber surface [35,36]. Similar symptoms were observed in our research for *F. sambucinum* and *F. oxysporum*. Their growth was visible as white, wadded mycelial and spore pads on the tubers, which partially collapsed (Table 1). Other fungal pathogens of potatoes are *Alternaria* species, the cause of early blight. This disease manifests as sunken, dark lesions on the tuber surface [37]. In the present work, *A. tenuissima* caused the most substantial infectious changes among the three tested *Alternaria* strains (Table 1). White and light brown, lumpy mycelial and spore pads were observed on the whole surface of the potato and the tubers were also sunken. *A. alternata* and *A. solani* induced only poorly visible white mycelial spots on the potato tubers. The symptoms of *Phoma exigua*, the cause of gangrene, are most often described as sunken, thumbprint-like lesions on the potato surface. Inside the tuber, dark rot develops [38]. In our experiments, a rotting and lumpy mass was observed inside the potato tubers (Table 1). Black scurf is another fungal disease of potato, caused by *Rhizoctonia solani*, and dry ulcer rot is the most common symptom of this disease [39]. Foaming, decaying mass inside and slightly visible white, wadded mycelium on the tuber surface was noted in our work (Table 1). In addition, *Colletotrichum coccodes* (black dot disease) can infect potato tubers during storage. The crop damage caused by this pathogen is increasing. The symptoms of infestation include silvery lesions on the tuber surface and the presence of black microsclerotia [9]. In our research, however, potatoes infected with *C. coccodes* did not reflect the literature description. This pathogen-induced tuber decay, formed a soft mass, clearly separated from the skin (Table 1). Differences between the laboratory method and naturally occurring way of pathogen inoculation may be the reason for this. One of the most serious bacterial diseases of potato is soft rot, caused by *Pectobacterium carotovorum*. Sunken lesions and decayed inside of the tuber are reported as the main symptoms of this disease [40], and the same tuber changes were observed in our experiments. A cream-colored, soft mass with a characteristic smell occurred after incubation of tubers infected with *P. carotovorum* (Table 1). This bacterium (as well as *Pectobacterium versatile* and *Pectobacterium atrosepticum*) is also responsible for blackleg disease that causes wilting of the whole plant, and blackening and necrosis of the stem [41]. Another significant bacterial pathogen of potato is *Streptomyces scabiei*, the cause of common scab. This disease manifests as a pitted, erumpent, or mild netted scab of potato tuber [40]. Small, brown scabs were also observed in our experiments (Table 1). 

However, such disease symptoms may not be visualized until the infection has progressed significantly, and it is often too late to implement disease mitigation measures. In addition, there could be discrepancies and subjectivity involved in human inspections. Furthermore, visual approaches may be destructive and render limited sampling accuracies [42]. 

As the result of data analysis obtained from HS-SPME-GC-MS measurements, a total of 130 compounds were identified. The detailed results of these findings are summarized in Table S1. The results were expressed as means of peak area % from duplicate analysis of each sample. The relationships among different phytopathogens used to contaminate potato tubers and volatile compounds after 14 days of storage were determined using a PCA analysis. The principal component analysis (PCA) was carried out using the relative content (% peak area) of volatile compounds as variables. A double criterion was applied to estimate the number of PCA factors that significantly affect the total variance: the own value chart and own values > 1. Using the above criteria, the four PC factors were identified, which explained 67.26% of the total variance.

**Figure 1:**
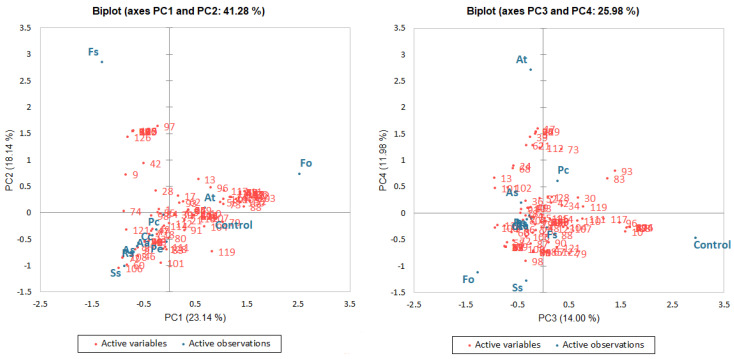
Principal component analysis (PCA) biplot of volatile compounds determined in potato tuber samples contaminated with different phytopathogens. Fo—*F. oxysporum*; Fs—*F. sambucinum*; Aa—*A. alternata*; As—*A. solani*; At—*A. tenuissima*; Rs—*R. solani*; Cc—*C. coccodes*; Pe—*P. exigua*; Ss—*S. scabiei*; Pc—*P. carotovorum*.

The scatter score plot of principal components (Figure 1) showed the distinction between contaminated samples and the relationship between phytopathogens and volatile compounds determined after 14-day potato storage. The first two factors (PC1 and PC2) accounted for 41.28% of the total variance. The first component (PC1) was characterized by 37 compounds, mainly alkanes. The second factor (PC2) was characterized by 31 compounds mostly including terpenes (i.e. α-cubebene, D-limonene, α-guaiene, spiro[3.4]octan-5-one, 3-carene, and chamigrene). Moreover, some alcohols (3-octanol and 3-methyl-1-butanol) and alkanes (1-methyl-4-propylbenzene, 1,2,4-trimethylbenzene, and methylbenzene), as well as dimethyl disulfide, showed high values in the second component. The last components (PC3 and PC4) were both characterized by 20 compounds. The third component accounted for about 14% of the total variance and correlated mainly with 1-butanol, 2-butanone, benzaldehyde, ethyl acetate, propylbenzene, isopropylbenzene, and undecane, whereas the fourth component accounted for 11.98% of the total variance and was related mainly to β-cedrene, 1-octen-3-ol, 2-nonen-1-ol, benzothiazole, and isobutylbenzene.

Moreover, during data analysis, the observations were classified based on their correlation to each PC factor (Table 2). The PCA was able to separate samples in the function of phytopathogens used for contamination. The PC1 factor is associated with three samples inoculated with *Alternaria solani*, *Fusarium oxysporum*, and *Rhizoctonia solani* strains; although, the *Fusarium oxysporum* strain shows the strongest relationship. Likewise, factors PC2 and PC4 are related to the three trials while the stronger relationship can be linked to the samples inoculated with *Fusarium sambucinum* and *Alternaria tenuissima* strains, respectively. In turn, the third factor (PC3) is related only to the control sample. 

**Figure 2:**
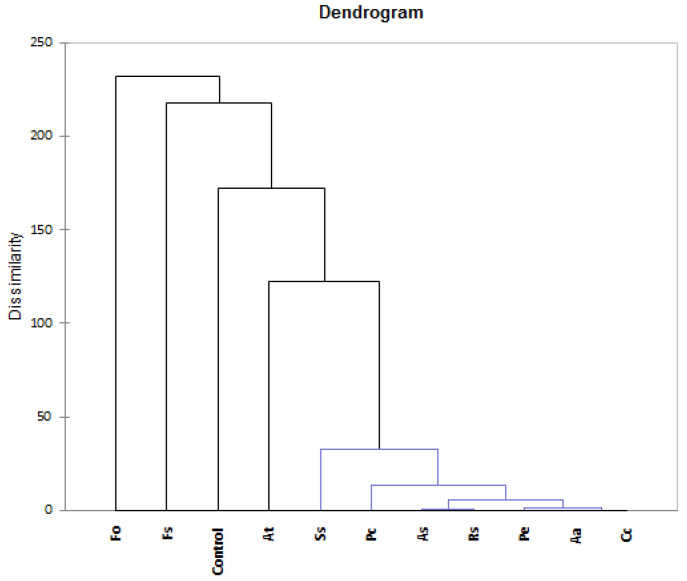
Dendrogram from the hierarchical clustering analysis (HCA). Fo—*F. oxysporum*; Fs—*F. sambucinum*; Aa—*A. alternata*; As—*A. solani*; At—*A. tenuissima*; Rs—*R. solani*; Cc—*C. coccodes*; Pe—*P. exigua*; Ss—*S. scabiei*; Pc—*P. carotovorum*.

The hierarchical cluster analysis was also applied to evaluate similarities between volatile compounds emitted from potato tuber samples contaminated with different phytopathogens. According to data presented in Figure 2, HCA divided samples into five groups. Groupings were made by using Ward’s method and squared Euclidean distance. The first cluster was represented only by the control sample, while the second cluster, in turn, contained seven samples contaminated with *A. alternata*, *A. solani*, *C. coccodes*, *P. carotovorum*, *P. exigua*, *R. solani*, and *S. scabiei.* The profile of these samples was dominated by 3-methyl-1-butanol, 2-methyl-1-butanol, D-limonene, α-pinene, β-pinene, acetic acid, 3-carene, p-cymene, heptanal, octanal, decanal, 3-methylbutanal, 2-methylbutanal, eucalyptol, 2-phenylisopropanol, and dibenzofurane. The last three clusters were represented by the samples contaminated with *A. tenuissima* (3rd), *F. oxysporum* (4th), and *F. sambucinum* (5th), respectively. The predominant compounds included: 1-octane-3-ol, benzothiazole, β-cedrene, isobuthylbenzene, and 1,2,3-trimethylbenzene (At), 1,2,3-trimethylcyclopentane, 1-ethyl-4-methylbenzene, 2-methylheptane, 3-methyloctane, 4-methyloctane, 4-heptanone (Fo), and 3-octanol, valencene, chamigrene, dimethyl sulfide, α-cubebene, α-guaiene, (+)-epi-bicyclosesquiphellandrene, and spirou[3.4]octen-5-one (Fs).

A characteristic group of compounds identified in the sample contaminated with *F. sambucinum* are sesquiterpenes such as valencene, α-cubebene, α-guaiene, chamigrene, and (+)-epi-bicyclosesquiphellandrene. Moreover, β-cedrene was detected only in the sample contaminated with A. tenuissima (Table S1 and Table 3). Jeleń and Wąsowicz [7] in an extensive review concerning the relationship between the spoilage of agricultural products and volatile metabolites of fungi cited the studies conducted by Zeringue et al. [43], which showed a correlation between the release of volatile compounds by Aspergillus flavus and *Aspergillus* fungi, including sesquiterpenes, and the initiation of mycotoxin biosynthesis. Shaw et al. [44], in turn, confirmed the ability of fungi to synthesize eucalyptol (1,8-cineole). In this study, eucalyptol was identified only in the sample contaminated with *S. scabiei* (Table 2 and Table 3). Another compound representing the terpene group is D-limonene, the monoterpene, which was found only in the contaminated samples, in the case of *A. alternata*, *C. coccodes*, *P. carotovorum*, and *S. scabiei*. In these samples, D-limonene was marked in the large relative content of 34.19, 20.18, 32.72, and 25.24% peak area, respectively (Table 3). D-limonene was commonly detected as a metabolite of fungi [7,44,45]. D-limonene and α-pinene identified for all the tested pathogens are regarded as the markers of potato phytotoxicity and have also been found to inhibit sprouting [46]. In turn, octanone reported for *C. coccodes* samples was previously detected in the early stages of potato storage [47]. 3-carene was also detected in several trials; however, compared to the control sample before storage, the highest relative content of this chemical was in the headspace from potato tubers contaminated with *A. solani* (9.14% of the total peak area). As in the case of D-limonene, no 3-carene was detected in the control sample after storage. 3-carene has also been recognized to have antimicrobial activities [48]. The studies of Ouellette et al. [49] confirmed the presence of dimethyl disulfide in the headspace of containers filled with potato tubers contaminated with two pathogens *Erwinia carotovora* var. *carotovora* and *Fusarium roseum* var. *sambucinum*. In this study, dimethyl disulfide was found in the sample infected with *F. sambucinum* (Table S1 and Table 3). Apart from terpenes, other fungal origin compounds were found, including hydrocarbons, acids, ketones, and alcohols [7,50,51,52,53,54]. In the samples inoculated with *C. coccodes* and *R. solani*, a methylated derivative of benzene, i.e., 1,2,4-trimethylbenzene was detected, which was not found in other samples, including the control. Production of various hydrocarbons by fungi has been reported previously [55]. 2,6,11-trimethyldodecane, in turn, was detected only in the sample contaminated with *A. tenuissima* (Table S1). In addition, benzothiazole was a volatile marker of the growth of *A. tenuissima* on potato tubers. De Lacy Costello et al. [45] noticed the largest peak of beznothiazole in the headspace of potato tubers infected with *Fusarium coeruleum* and *Phytophthora infestans*. From the observations, two alcohols, i.e., 3-octanol and 2-butanol can be associated with the growth of the phytopathogens namely *F. sambucinum* and *P. carotovorum*, respectively. The species of *Fusarium* have been identified to produce alcohols, which can be used as biomarkers for these fungi [6]. The acetic acid, in turn, was present in all infected samples; however, relative to the control sample before storage, its content increased from 2.04% of the total peak area to 19.97 (*R. solani*), 16.45 (*A. solani*), 11.91 (*S. scabiei*), 10.79 (*P. exigua*), 9.31 (*A. alternata*), and 7.54 (*C. coccodes*) % of total peak area. However, acetic acid was not detected after storing the control sample. Among ketones determined in the tested samples, spiro[3.4]octan-5-one (*F. sambucinum*), 3-octanone (*C. coccodes*), and 1-octen-3-one (*A. solani*) can be regarded as the markers of phytopathogen pattern on potato tubers. These organics were identified as volatile compounds produced by *Muscodor suthepensis* [56], *Alternaria alternata* [50], and *Rhizoctonia solani* and *Trichoderma viride* [57].

The undertaken study has shown a multiple of influences of the phytopathogens infecting seed potatoes on both emitted volatile organic compounds and individual parameters of plant growth, development, and physiological activity. The obtained results coincided in varying degrees and were often complimented, which indicates the necessity to use a wide range of tests to comprehensively investigate the effects of seed potato treating.

The positive or negative relationships between the growth kinetics of stems and roots and their physiological activities indicate the applicability of specific parameters as markers of the biological quality of seed potatoes exhibiting different physiological states. The seed potatoes’ quality could be linked to a variety of biochemical and physiological changes that are required to initiate germination processes followed by the growth of plants and their yielding potential. 

**Figure 3:**
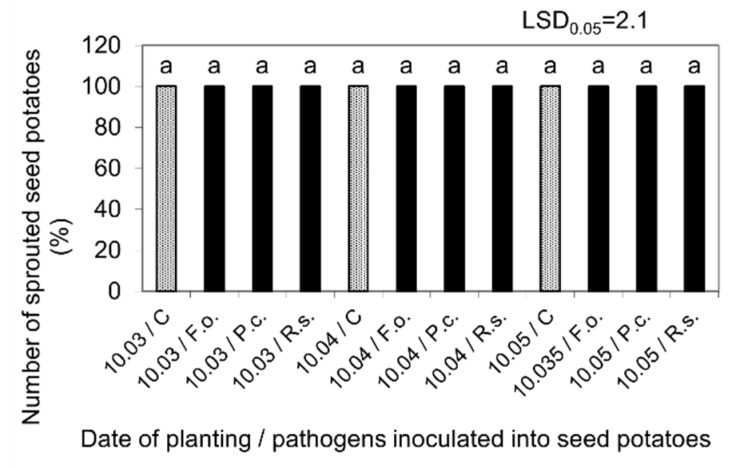
Percentage of the Impresja sprouted seed potatoes at 20 °C after storage at 4 °C from harvest until inoculation with phytopathogens on 10 February and then at 15 °C and 80% RH before planting in the soil on 10 March, 10 April, and 10 May. C—Control, Fo—*F. oxysporum*, Pc—*P. corotovorum*, Rs—*R. solani*. Means marked with the same letters do not differ statistically at the significance level *p* = 0.05. LSD was calculated at the significance level of *p* = 0.05.

**Figure 4:**
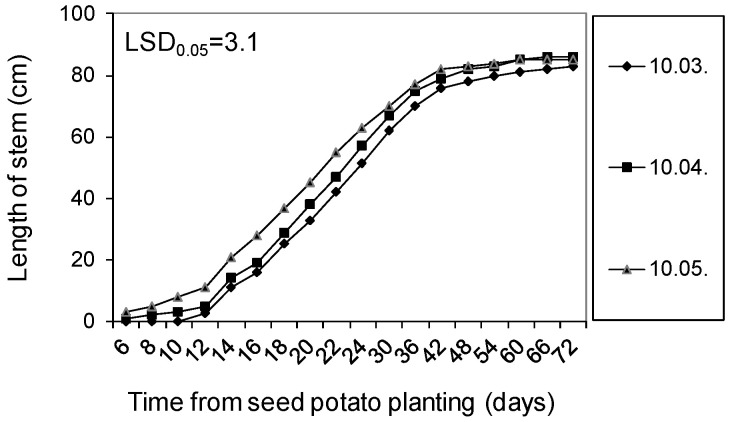
Growth kinetics of Impresja potato stems obtained from seed potatoes stored at 4 °C from harvest until 10 February of the following year and then at 15 °C and 80% RH until planting in the soil on 10 March, 10 April, and 10 May. LSD was calculated at the significance level of *p* = 0.05.

Untreated (control) seed potatoes stored at 4 °C from harvest until 10 February 2021, and then at 15 °C and 80% RH for the next three months until 10 May, were characterized by high health and typical phenotypic features. They germinated in 100% RH after planting in the substrate at 20 °C (Figure 3). During planting in the plant microcosms on 10 March, they had dormant buds, while when planting on 10 April and 10 May, they were already sprouted and produced sprouts with a length of approx. 2 and 5 cm, respectively. The plants obtained from them, in the performed three cultivation periods presented, started to emerge 6 days after planting of the seed potatoes and grew for the next 72 days, reaching the final height of 80–85 cm and forming flower buds. The plants obtained from sprouted seed potatoes planted on 10 April and 10 May, developed correspondingly earlier and faster than those grown from mother tubers planted in March with dormant buds (Figure 4).

**Figure 5:**
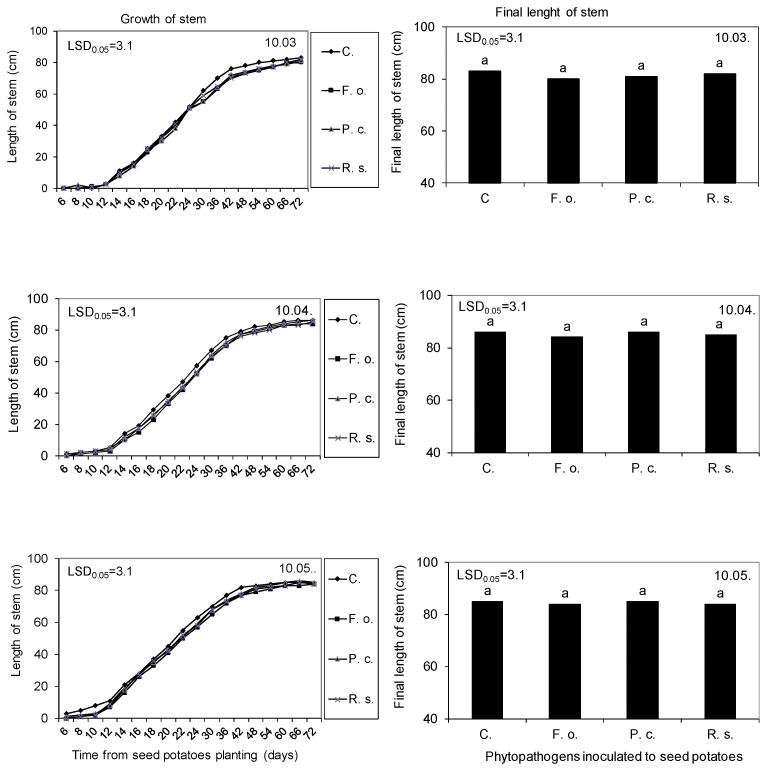
Growth kinetics and final length of Impresja stems obtained from the seed potatoes stored at 4 °C from harvest until inoculation with phytopathogens on 10 February and then at 15 °C and 80% RH before planting in the soil on 10 March, 10 April, and 10 May. C—Control, Fo—*F. oxysporum*, Pc—*P. corotovorum*, Rs—*R. solani*. Means marked with the same letters do not differ statistically at the significance level *p* = 0.05. LSD was calculated at the significance level of *p* = 0.05.

The inoculation of phytopathogens (*Fusarium oxysporum*, *Pectobacterium carotovorum*, and *Rhizoctonia solani*) to seed potatoes on 10 February had a varied effect on plant development, depending on the time elapsed from pathogen inoculation to plant emergence and also on sprouting stage during the planting of mother tubers into the soil. During planting in plant microcosms, the seed potatoes and the sprouts showed neither mechanical damage nor visible symptoms of disease infestation. Stems, obtained from the seed potatoes inoculated with phytopathogens, developed slightly slower than in control variants without adverse influence on flowering. These relationships occurred during cultivation in the three cycles applied; although, all stems and roots obtained from seed potatoes sprouted and planted in May developed the fastest in contrast to the ones planted in April and March (Figure 5).

**Figure 6:**
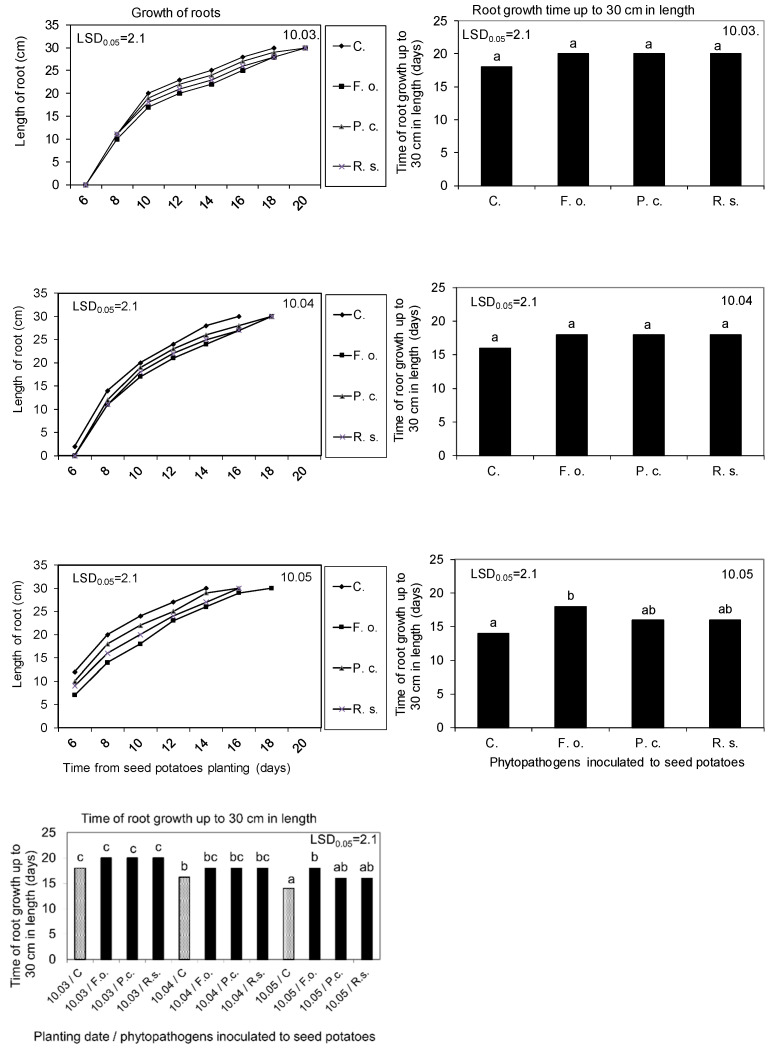
Kinetics of root growth and time of its growth up to 30 cm in length, as affected by the seed potatoes storage at 4 °C from harvest until inoculation with phytopathogens on 10 February and then at 15 °C and 80% RH before planting in the soil on 10 March, 10 April, and 10 May. C—Control, Fo—*F. oxysporum*, Pc—*P. corotovorum*, Rs—*R. solani*. Means marked with the same letters do not differ statistically at the significance level *p* = 0.05. LSD was calculated at the significance level of *p* = 0.05.

**Figure 7:**
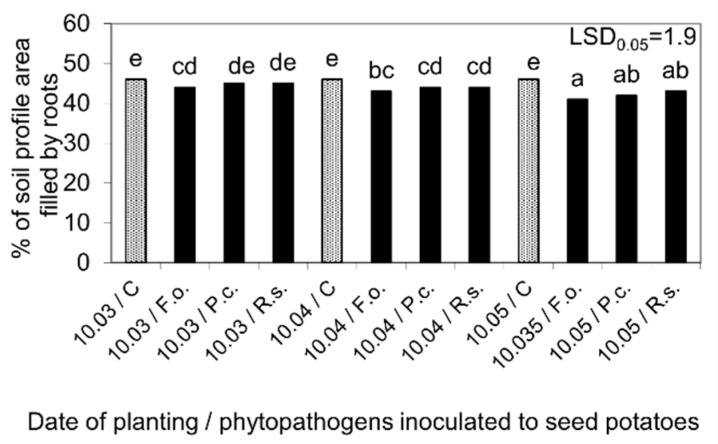
Percentage of the soil profile area filled by the roots 72 days after planting the seed potatoes into the ground, as affected by their storage at 4 °C from harvest until inoculation with phytopathogens on 10 February and then at 15 °C and 80% RH before planting in the soil on 10 March, 10 April, and 10 May. C—Control; Fo—*F. oxysporum*, Pc—*P. corotovorum*, Rs—*R. solani*. Means marked with the same letters do not differ statistically at the significance level *p* = 0.05. LSD was calculated at the significance level of *p* = 0.05.

The inoculation of seed potatoes with phytopathogens on 10 February had a more spectacular and negative effect on the kinetics of root growth, assessed on the basis of particular root lengthening and the overgrowth (filling) of the soil profile with the entire root system. In the control variant, non-sprouted seed potatoes planted on 10 March developed roots at the latest. Planting seed potatoes at later dates, when they had sprouts of about 2 (10.04) and 5 cm (10.05), resulted in a gradual acceleration of the root system development. As a result, it took 18, 16, and 14 days for the roots to grow up to a final length of 30 cm when the seed potatoes were planted in March, April, and May, respectively. Compared to the control variant, the infestation of seed potatoes with phytopathogens delayed the beginning of root growth, slowed down the kinetics of their development, and lengthened their growing period to a length of 30 cm. This delay was the lowest for the first planting date (10 March), when the period from phytopathogen inoculation to seed potato planting was the shortest and the buds showed no growth. In the case of subsequent cultivation dates, the negative impact of inoculated phytopathogens on the particular root growth and development of the whole root system was gradually increased. As a result, the dynamic of root growth under the influence of tested phytopathogens inoculation was the lowest after planting seed potatoes on 10 May, compared to the control. The longer time from inoculation up to seed potatoes planting also exhibited greater differences in the degree of the negative impact of *Fusarium oxysporum*, *Rhizoctonia solani*, and *Pectobacterium corotovorum* on the root system development, with *Fusarium oxysporum* being the most pathogenic species (Figure 6 and Figure 7).

**Figure 8:**
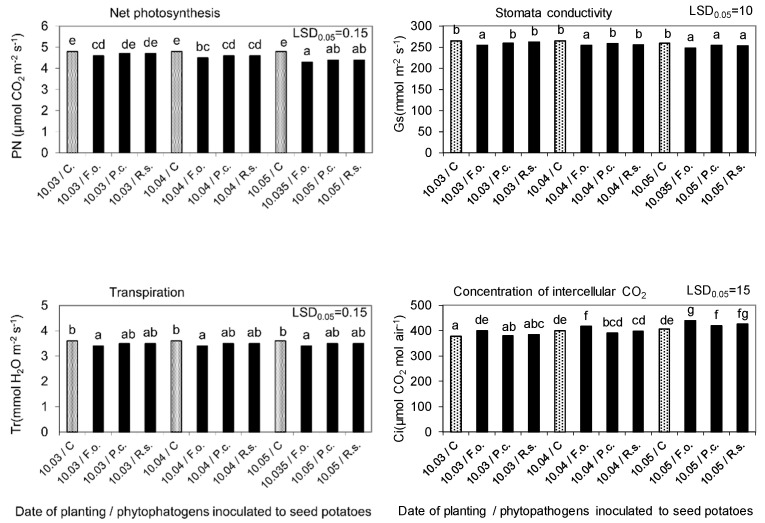
Gas exchange in potato leaves, as affected by seed potatoes storage at 4 °C from harvest until inoculation with phytopathogens on 10 February and then at 15 °C and 80% RH before planting in the soil on 10 March, 10 April, and 10 May. C—Control, Fo—*F. oxysporum*, Pc—P*. corotovorum*, Rs—*R. solani*. Means marked with the same letters do not differ statistically at the significance level *p* = 0.05. LSD was calculated at the significance level of *p* = 0.05.

**Figure 9:**
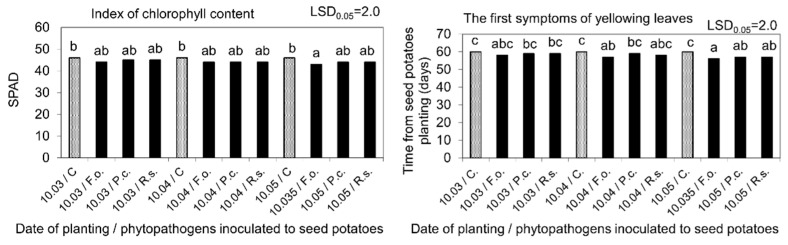
Index of chlorophyll content and first symptoms of their yellowing, as affected by seed potatoes storage at 4 °C from harvest until inoculation with phytopathogens on 10 February and then at 15 °C and 80% RH before planting in the soil on 10 March, 10 April, and 10 May. C—Control, Fo—*F. oxysporum*, Pc—*P. corotovorum*, Rs—*R. solani*. Means marked with the same letters do not differ statistically at the significance level *p* = 0.05. LSD was calculated at the significance level of *p* = 0.05.

Phytopathogens inoculated into seed potatoes also showed a negative effect, to a varying degree, on the physiological activity of plants assessed based on measurements of gas exchange in the leaves (net photosynthesis, transpiration, stomata conductivity, and intercellular CO_2_ content) and the index of chlorophyll content indicating the content of this photosynthetic dye. The changes in physiological activity demonstrated by these markers were comparable to the parameters of the biometric evaluation of stems and roots. An unfavorable effect of phytopathogens was mostly evident after seed potatoes planting on 10 May, i.e., when their storage period at 15 °C and 80% RH after inoculation was the longest (Figure 8 and Figure 9). 

The inoculated phytopathogens, in most cases, did not show a significant negative effect on the quality of stems and foliage, assessed using a five-point valuation scale; although, in all cases, there were trends of their negative impact. Most of the plants were similarly colored and changed their color from green to yellow after a similar time from the beginning of emergence, with the exception of those obtained from seed potatoes planted on 10 May, which started to change it earlier than in the other experimental variants.

Phytopathogens applied to seed potatoes also exhibited a negative effect on the yield of tubers and stems. In the control variant, the yield of the fresh and dry weight of tubers and stems was significantly higher, as the more sprouted seed potatoes were planted in the substrate at a later date, with the more advanced development of sprouts. Infection of seed potatoes with phytopathogens and planting them on the first date (10.03) caused a slight, often insignificant decrease in the yield of tubers and stems compared to the control. On the other hand, a longer period from the moment of infection with phytopathogens to the time of planting at a later date (10 April and 10 May) resulted in a gradual reduction in the yield of fresh and dry tuber and stem biomass (Table 4). This was most likely due to the less advanced infestation by phytopathogens of the sprouts grown from seed potatoes planted in the ground shortly after inoculation. Storage of inoculated seed potatoes until 10 May at 15 °C and 80% relative humidity was conducive to contamination of the entire slowly developing sprouts by the pathogenic mycoflora. It facilitated the infection of the stems and roots grown on these sprouts, which resulted in their slower development and lower tuber yield. 

The performed analyzes showed negative effects of the tested phytopathogens on the growth and quality of plants, gas exchange, index of chlorophyll content, and the fresh and dry mass of tubers and dried stems. However, the demonstration of the magnitude of the negative impact of these pathogens and volatile compounds depended on the marker tested. The most useful markers were the ones that validated the growth and development of the root system, gas exchange, and the yield of tubers, and exhibited less growth of stems and the index of chlorophyll content. Most likely, this might depend on the distance of the inoculated tissue from the growing shoots, roots, and stems, and on the metabolism and movement of pathogens to individual plant organs. This indicates that a comprehensive analysis of the effects of various seed potato treatments, including pathogen infestation, requires the use of all the markers tested, which together indicate the overall plant development. On the basis of all these parameters, it is possible to determine the health of seed potatoes and forecast the development of plants obtained from them in field conditions, which was confirmed by our previous research on several other crops [14,15,16,19,20,21,22]. 

A quick examination of the effect of phytopathogens was possible thanks to the use of elaborated plant microcosms, which, in a short time, enabled to trace the entire development cycle of individual plant organs before planting the seed potatoes in the field [17,18]. The markers used for the evaluation of the potato development in plant microcosms demonstrated, in a short time period, the high sowing value of seed potatoes, which were planted on 10 March, 10 April, and 10 May. They exhibited that the stems and roots obtained from the more sprouted seed potatoes and those planted later developed faster and produced a higher yield of fresh and dry biomass of tubers. As a result, the plants obtained from the sprouted seed potatoes planted in the substrate in May, with sprouts approximately 5 cm long, developed the fastest and gave the highest yield. The analyzed data are consistent with the research of Rykaczewska [68], in which the storage of seed potatoes in similar conditions until April had a positive effect on the growth of stems. They are also consistent with the findings of Stumm et al. [69] who studied only the stem growth from seed potatoes stored by various methods. According to the Research Centre for Cultivar Testing in Poland [70], the yield of potato tubers of the Impresja cv. 40 days after emergence was 0.44 kg per plant with a spacing of 50,000 plants per ha and 0.49 kg with 45,000 plants per ha, hence, similar to the crop used here in plant microcosms.

The conducted research demonstrated that the most useful and quick marker of seed potato infection by phytopathogens was the kinetics of root growth and development, enabled to perform in plant microcosms. The intensity of growth kinetics of both individual roots and the entire root system, evaluated on the basis of their filling of the soil profile, was strictly dependent on the degree of seed potato infestation by phytopathogens, which was increased when the period from inoculation to tuber planting was longer. The usefulness of this marker was high because the development and physiological activity of roots, closest to sprout eyes, were earlier than of stems, and it was dependent on the health and yielding potential of seed potatoes. Moreover, roots determine to a large extent the stems’ growth, nutrients, and water uptake, as well as vigor, quality, and yielding of plants. The dynamics of root growth can also be a valuable and quick-to-perform marker of plant development in the case of contamination by phytopathogens deriving from the soil. Root growth kinetics also showed, to a greater extent than other markers, the degree of pathogenicity of phytopathogens inoculated to sprouted seed potatoes. By using this marker, it was also demonstrated that all the tested phytopathogens, i.e., *Fusarium oxysporum*, *Rhizoctonia solani*, and *Pectobacterium corotovorum*, had a negative effect on the growth, development, and yielding of plants, while *Fusarium oxysporum* exhibited a greater infection potential. This was most evident in the case of sprouted seed potatoes planted in the ground on 10 May, three months after inoculation. A detailed understanding of this issue needs further research. 

Stem growth kinetics as a marker of the quality of seed potatoes infested with phytopathogens was also useful. However, the negative effect of phytopathogens inoculated into seed potatoes and the emitting volatile compounds on stem growth and quality was less spectacular than in the case of root development; although, in most experimental variants there was a trend or statistically proven evidence of an adverse effect of these microorganisms on the development of above-ground plant organs. 

The used markers showed that the tested phytopathogens inoculated into seed potatoes also influenced, to a different extent, the intensity of gas exchange (net photosynthesis, transpiration, stomatal conductivity, and intercellular CO_2_ content), index of chlorophyll content, tuber and stem yield, and the quality of foliage measured on a five-point valuation scale. These parameters may serve as useful markers of the quality of seed potatoes infected with phytopathogens; although, their evaluation requires a longer plant cultivation period than in the case of root growth kinetics.

All the used markers showed that unfavorable effects of phytopathogens were relatively the smallest in the case of plants obtained from seed potatoes less colonized by fungi and planted in the ground on 10 March, i.e., 30 days after inoculation. They were more evident in the case of plants obtained from seed potatoes that were sprouted two months after inoculation and planted into the ground on 10 April, and even greater when they were planted on 10 March, three months after inoculation. This indicates that a longer period from the inoculation of phytopathogens until the seed potatoes planting had a more negative effect on plant development. In the shorter time, these pathogens probably only slightly developed, which resulted in their less negative impact on plant development.

The less spectacular effect of phytopathogens on the stem development, demonstrated by the markers used, could be due to their limited pathogenic activity after inoculation to seed potatoes and their lower infectious possibility in conditions of very rapid root and stem growth in favorable micro-environmental conditions, as well as the possibility of intensive regenerative processes of infected tissues. It could also result from minor damage of the seed potato tissues, which in total prevented a more thorough penetration of the applied fungi into seed potatoes and resulted in a lower infestation. The slightly infected seed potatoes exhibited a greater resistance to phytopathogens because they were characterized by a high yielding potential, germinated quickly in 100% at 15 °C and 80% RH, and the plants obtained from them emerged 6 days after mother tuber planting in the substrate, whereas in field, they usually emerged after 12 days. Moreover, the tested phytopathogens were inoculated into the crumb of the seed potatoes and their displacement into the fast-growing roots and the stems, perhaps not being fast enough to infect these organs. In this regard, longer storage of sprouted seed potatoes after inoculation of phytopathogens could favor greater infection with them of the sprouts, roots, and stems, which would result in slower plant growth. In addition, the rapidly increasing assimilation surface area of leaves in the spring and summer period favored a greater use of photosynthetically active radiation reaching plants, and thus increased the intensity and productivity of photosynthesis, as well as plant resistance to infections and stress [71,72]. The obtained results refer to the results of previous own research, in which, in turn, phytopathogens inoculated into more damaged internal tissues of seed potatoes caused their severe infection and death, which prevented the sprouting and growth of sprouts.

The results show that despite some of the data presented in the literature, it is still essential to increase the knowledge concerning rapid detection and signaling changes in seed potatoes sowing value by means of physiological markers necessary for use in optimizing the plant treatment and increasing their health. Their potential advantages may allow the tests to be widely used in many laboratories as environmentally friendly. Therefore, the investigated physiological parameters of the seed potato quality can be applied as markers of their germinability, health, and sowing value. 

The presented findings expanded the existing knowledge on the pathogenicity of inoculated phytopathogens into seed potatoes, cv. Impresja and the emitted volatile organic compounds. They also demonstrated the possibility of a comprehensive study of phytopathogen influence on development, physiological activity, and yielding of plants, using the developed markers, whose indications coincide or complement.

## 3. Materials and Methods

### 3.1. Potato Pathogens

Two bacterial and eight fungal potato pathogens were used in the research. All the strains are listed in Table 5.

Bacteria strains were activated on Tryptic Soy Agar—TSA (Merck, Darmstadt, Germany) while fungal strains on Potato Dextrose Agar—PDA (Merck, Darmstadt, Germany). Plates were stored at 4 °C. Pathogen suspensions were prepared from the pure cultures on the TSA/PDA agar plates and adjusted to a final concentration of 10^6^ CFU/mL in 0.85% NaCl.

### 3.2. Seed Potatoes Inoculation with Phytopathogens

Seed potatoes cv. Impresja were obtained from Zamarte Potato Breeding (Zamarte, Poland). Potatoes were rinsed with distilled water and left to dry. Then, the cuts with a sterile scalpel in the shape of an X (1 cm depth, 2 cm width) were performed. Seed potatoes were immersed with the freshly prepared phytopathogens’ suspensions. The visual assessment was conducted for all tested pathogens after 14 days of incubation at 25 °C and 80% RH. 

Volatile compounds were analyzed for potato samples inoculated with each pathogen. For this purpose, containers were filled with 650 g ± 5 g (8 pieces) of seed potatoes and incubated at 15 °C and 80% RH for 14 days. The HS-SPME-GC analysis was performed at the beginning of the incubation period and after it was finished. The control sample was not inoculated with the phytopathogens. 

For growth and physiological parameters analysis of infected seed potatoes, *P. carotovorum, R. solani,* and *F. oxysporum* fungi were selected as the most significant potato pathogens as being responsible for huge economic losses in potato production. Seed potatoes were inoculated with pathogens’ suspensions on 10 February. Then, the samples were incubated at 15 °C and 80% RH until 10 March, 10 April, and 10 May.

### 3.3. Analysis of Volatile Compounds (HS-SPME-GC)

Volatile compounds were analyzed using an Agilent 7890A chromatograph (Agilent Technologies, Santa Clara, CA, USA) coupled to a mass spectrometer Agilent MSD 5975C (Agilent Technologies, Santa Clara, CA, USA). A capillary column DB-1ms 60 m × 0.25 mm × 0.25 µm (Agilent Technologies Santa Clara, CA, USA) was applied to separate the compounds. For extraction of volatile compounds, the solid-phase microextraction technique was used. Adsorption was carried out by using the fiber covered with 50/30 μm Divinylbenzene/Carboxen/Polydimethylsiloxane (DVB/CAR/PDMS) phase (length 1 cm). 

The glass vessel with potatoes (i.e., control samples, as well as contaminated samples before and after 14-day storage), was tightly covered with a lid containing a sampling port and then incubated at 20 °C for 30 min. During this time the vessel was saturated with volatile compounds from potato tubers. Next, the SPME fiber was inserted into the headspace of the vessel via the sampling port, followed by the exposure for 60 min at 20 °C. After adsorption of volatiles from the headspace, the fiber was retracted into the needle and transferred to the inlet of the GC apparatus for desorption of volatile analytes. Desorption was carried out for 5 min at 250 °C. For fiber cleaning before each extraction, the fiber was heated for 10 min in the inlet of the GC apparatus, at 260 °C.

All injections were performed in a splitless mode. As a carrier gas, helium was used with a flow rate of 1.1 mL/min. The GC oven temperature was programmed to increase from 30 °C (10 min) to 70 °C at a rate of 5 °C/min and kept for 1 min, then to 230 °C at a rate of 10 °C/min, and finally kept for 2 min. The temperatures of the MS ion source, transfer line, and quadrupole analyzer were 230, 250, and 150 °C, respectively. The electron impact energy was set at 70 eV. The mass spectrometer was operating in the full scan mode. Qualification of volatile compounds was performed by comparison of obtained spectra with the reference mass spectra from NIST/EPA/NIH mass spectra library (2012; Version 2.0 g.) and confirmed with the use of the deconvolution procedure. Moreover, retention indices (RI) were calculated according to the formula proposed by van den Dool and Kratz [73] relative to a homologous series of n-alkanes from C5 to C23. Retention indices were compared with reference compounds and literature data [74,75]. Data processing was conducted with Mass Hunter Workstation Software (Agilent, Santa Clara, CA, USA). The control sample as well as infected samples were prepared in duplicate.

### 3.4. Growth and Physiological Parameters Analysis

#### 3.4.1. Methods of Plant Cultivation

After incubation, potatoes were subjected to negative selection and then cultivated in a ventilated greenhouse, in plant microcosms (30 × 40 × 10 cm: height × width × depth; front walls from transparent glass), which simulated the field conditions and additionally enabled quick evaluation of the development, health, and physiological activity of stems and root system, as well as changes in soil structure [17,18]. Microcosms were filled with 10 L of standard substrate, which was enriched with fertilizer containing macro- and microelements (YaraMila Complex; Yara) at a dose of 2 kg/m^3^. In each experimental variant, plant microcosms were set up in triplicate in random blocks. The experiments were carried out in Skierniewice, Central Poland (51°57′010″ N, 20°08′030″ E), where the temperature in July fluctuates from 8 to 32 °C and the average annual precipitation is 528.3 mm. However, the temperature in greenhouse was set at 20–25 °C, the number of sunny days was 8.7–11.6 h per day in April–July and the plants were watered with tap water when needed. During cultivation, the plants were periodically sprayed, as needed, with the ecological preparation Limocide (Agrosimex; insecticide, micro-emulsion acaricide) at a dose of 4 L/ha in order to protect them against insects, mainly against the greenhouse whitefly and the Colorado potato beetle.

#### 3.4.2. Assessment of Growth and Physiological Activity Parameters

Seed potatoes were observed every day since pathogen inoculation and during storage. After their planting in substrate in plant microcosms, the growth and physiological activity of the plants were observed every 2 days for the first 24 days and every 6 days for the remaining period. The following parameters were measured:

1. Biological condition of the seed potatoes, in terms of their vigor, turgor, rotting, and infection by phytopathogens;
2. Percentage of germinating tubers;
3. Kinetics of plant growth, by measuring the stem height every 2 days during the first 24 days and every 6 days for the remaining period [17,18,21];
4. Quality of the stems on a 5-point valuation scale, where 5 indicates stems full of vigor and well growing, and 1 indicates dried out ones [15,16];
5. Kinetics of root growth, by measuring its length every 2 days until it reaches 30 cm [17,18];
6. Kinetics of root system development, by assessing every 2 days the percentage of the soil profile area filled by the roots;
7. Intensity of gas exchange (net photosynthesis, transpiration, stomata conductivity, and intercellular CO_2_ content), measured with a TPS-2 apparatus (PP Systems, USA) in the highest positioned fully developed leaves, during the period of most dynamic plant growth [16];
8. Index of chlorophyll content, measured with a Minolta SPAD-502 apparatus (Japan) in the highest positioned fully developed leaves, during the period of most dynamic plant growth [16];
9. Fresh and dry (dried at 130 °C for 3 days) mass of tubers and withered stalks (haulms) at the end of the cultivation period [20,22];
10. Infestation of seed potatoes, stems, and roots by phytopathogens, assessed throughout the entire plant growth period [14].

### 3.5. Statistical Analysis

The results concerning plant growth and physiological activity were statistically evaluated using the analysis of variance (STATISTICA, version 10). Differences between the means were estimated using Duncan’s t-test at a significance level of 5.

Principle component analysis (PCA) and Hierarchical cluster analysis (HCA) were applied to test for significant differences between samples and for determination of the relatives between volatile compounds and testing strains used as tuber contaminants. The statistical analysis was performed using XLSTAT software (Addinsoft, version 2022.2.1, New York, USA). 

## 4. Conclusions

Pathogens emitted volatile organic compounds, and their activity had a significant impact on the germination and yielding potential of potatoes. The established markers enabled us to accurately demonstrate the negative effect of phytopathogens infecting seed potatoes not only on the kinetics of stem and root growth and the development of the entire root system, but also on gas exchange, and chlorophyll content in leaves and yield. The negative effect of phytopathogens was relatively low on the growth of plants obtained from seed potatoes planted in the substrate one month after infection, but it was much greater on plants obtained from seed potatoes grown for two months after infection and planted, and the greatest when they were planted three months after infection. All investigated fungal and bacterial phytopathogens, i.e., *Fusarium oxysporum, Rhizoctonia solani,* and *Pectobacterium corotovorum* showed an unfavorable effect on plant development, and *Fusarium oxysporum* turned out to be the most pathogenic. It was documented that different sesquiterpenes: dimethyl disulfide; 1,2,4-trimethylbenzene; 2,6,11-trimethyldodecane; benzothiazole; 3-octanol; and 2-butanol, were associated with the growth of *F. sambucinum, A. tenuissima,* and *P. carotovorum*. However, another metabolite—acetic acid—was detected in all infected samples. The research showed different usefulness of markers applied to show the toxic effect of inoculated phytopathogens on various stages of plant development and their individual organs. This justifies the need to use the proposed spectrum of tested markers, which enables a comprehensive assessment of the impact of pathogenic microorganisms on the entire development of potato plants. However, this is the first analysis to detect metabolites and indicate them as biomarkers. Markers should be validated in the future under set conditions, comparing two research methods, e.g., GC-MS and molecular analysis. The sensitivity and specificity analysis using the ROC curve could be used as a valuable tool for recognizing selected metabolites as biomarkers. Such studies should be carried out in the future. Collectively, this study may provide new insight into analytical methods to improve potato evaluation, opening a potential path toward various environmental technologies. However, the widespread use of volatility profiles in potato seed quality management practices remains a challenge, still largely linked to costly analytical procedures.

## Figures and Tables

**Table 1:** Visual assessment of the seed potatoes infestation with phytopathogens.

Photograph	Description	Photograph	Description
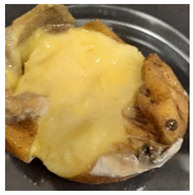	***Pectobacterium carotovorum*** Soft, cream-colored, decaying mass with a characteristic smell.	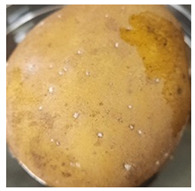	***Alternaria solani*** White mycelium spots on the whole surface.
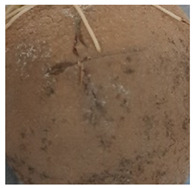	***Streptomyces scabiei*** Small, brown scab on the surface.	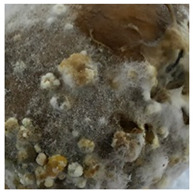	***Alternaria tenuissima*** White or brown, lumpy mycelium on the surface. The tuber collapsed.
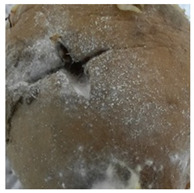	***Fusarium oxysporum*** White, wadded mycelial and spore pads on the surface. Tuber sunken.	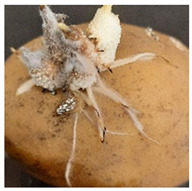	***Alternaria alternata*** White mycelium visible on the sprouts.
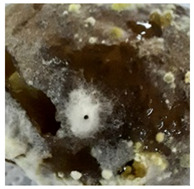	***Fusarium sambucinum*** White or yellowish-white, wadded mycelial and spore pads on the surface.	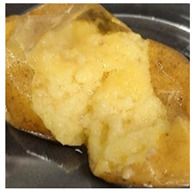	***Phoma exigua*** Rotting, lumpy mass. Skin clearly separated from the tuber interior.
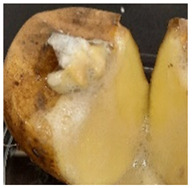	***Rhizoctonia solani*** White, wadded mycelium on the surface. Partially soft, foaming mass.	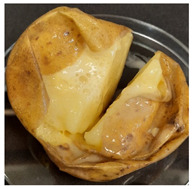	***Colletotrichum coccodes*** Soft mass, clearly separated from the tuber skin.

**Table 2:** Squared cosines of the observations.

Sample	PC 1	PC 2	PC 3	PC 4
Control	0.128	0.001	0.840	0.018
*A. alternata*	0.048	0.070	0.015	0.003
*A. solani*	0.120	0.087	0.037	0.006
*A. tenuissima*	0.093	0.001	0.007	0.751
*C. coccodes*	0.041	0.051	0.038	0.005
*F. oxysporum*	0.752	0.050	0.115	0.078
*F. sambucinum*	0.206	0.783	0.000	0.005
*P. carotovorum*	0.005	0.001	0.018	0.073
*P. exigua*	0.001	0.084	0.014	0.000
*R. solani*	0.147	0.120	0.014	0.001
*S. scabiei*	0.154	0.162	0.013	0.174

**Table 3:** Volatile compounds as markers of potato pathogens infestation.

Marker of Pathogen Infestation	Compound Name	Origin * (M/P)
All pathogens: *F. oxysporum*; *F. sambucinum*; *A. alternata*; *A. solani*; A. tenuissima; *R. solani*; *C. coccodes*; *P. exigua*; *S. scabiei*; *P. carotovorum*.	Acetic acid (no. 60)	M [7,45,50,58]; P [58]
	α-Pinene (no. 128)	M [7,44,52,59]; P [58]
	1-Methyl-3-propylbenzene (no. 13; with exception of *A. alternata*),	M [60]
	Decanal (no. 72; with exception of *P. carotovorum*)	M [7]
	Methylbenzene (no. 101)	P [58,61]
	Nonanal (no. 106)	M [45]
	p-Xylene (no. 119)	M [7,58]; P [58]
	p-Cymene (no. 111)	M [7]
*F. oxysporum*	2-Methylheptane (no. 35) 2-Methylhexane (no. 36) 3-Methylhexane (no. 52) 1-Ethyl-4-methylbenzene (no. 12) Cyclohexane (no. 68) Cyclohexanone (no. 69) 2,4-Dimethylhexane (no. 24) 4-Heptanone (no. 57) 1,2,3,-Trimethylcyclopentane (no. 6) 3-Methyloctane (no. 53)	M [53] M [61] M [61] ^#^ M [61] M [35] M [7] M [50] ^#^ M [61]
*F. sambucinum*	Valencene (no. 125) 3-Octanol (no. 54) α-Cubebene (no. 126) α-Guaiene (no. 127) Spiro[3.4]octan-5-one (no. 120) Chamigrene (no. 66) (+)-epi-Bicyclosesquiphellandrene (no. 2) Naphtalene (no. 105) Acenaphtene (no. 59) Dimethyl disulfide (no. 77) 1-Methyl-4-propylbenzene (no. 14) 1-Methylnaphtalene (no. 15)	M [7] M [7] M [7] M [7] M [56] M [7,51] M [7,43] M [7] M [62] M [5,7,63,64,65]; P [58] ^#^ M [52,65]
*A. alternata*	D-Limonene (no. 78) Pentane (no. 114) Cyclohexane (no. 68)	M [7,44,45,61]; P [58] M [7,49] M [61]
*A. solani*	1-Octen-3-one (no. 18) 3-Carene (no. 36)	M [36,57] M [35]
*A. tenuissima*	Isobutylbenzene (no. 96) 2,6,11-Trimethyldodecane (no.26) Benzothiazole (no. 64) β-Cedrene (no. 129)	^#^ M [53] M [45]; P [58] M [64]
*R. solani*	1,2,4-Trimethylbenzene (no. 7)	M [7,52]; P [58]
*C. coccodes*	D-Limonene (no. 78) 3-Octanone (no. 55) 4-Methyloctane (no. 58) 1,2,4-Trimethylbenzene (no. 7)	M [7,45,46,61]; P [58] M [7,50,51,53] M [51] M [7,52]; P [58]
*P. exigua*	2-ethyl-1,4-dimethylbenzene (no. 21) 1,2,4-Trimethylcyclopentane (no. 8) 2-Hexanone (no. 33)	M [60] ^#^ M [63]
*S. scabiei*	2-Methylbutanal (no. 51) 3-Methylbutanal (no. 50) Eucalyptol (no. 86) 2-Phenylisopropanol (no. 43) Cyclopentanone (no. 70) Hexadecane (no. 89) D-Limonene (no. 78)	M [50,52]; P [58] M [50,52]; P [58] M [7,44] M [66] M [7]; P [7] M [45,52]; P [7] M [7,44,45,61]; P [58]
*P. carotovorum*	D-Limonene (no. 78) 2-Butanol (no. 28)	M [7,44,45,61]; P [58] M [7]; P [58]
Present only in control sample, not infected with pathogens	1-Butanol (no. 10) 2,6-Dimethylundecane (no. 27) 2-Butanone (no. 29) 2-Pentanone (no. 41) Benzaldehyde (no. 61) Ethyl acetate (no. 81) Undecane (no. 124)	M [53]; P [58] M [53] M [56]; P [58,67] M [7,50,63]; P [61] M [7,52,61] M [7] M [53]; P [58]

All compound numbers refer to the order in Table S1. * M—microbial, P—plant; ^#^ unknow origin.

**Table 4:** Fresh and dry weight of tubers and dried stems per plant, obtained from the seed potatoes stored at 4 °C from harvest until inoculation with phytopathogens on 10 February and then at 15 °C and 80% RH before planting in the soil on 10 March, 10 April, and 10 May.

Date of Seed Potatoes Planting to Soil	Phytopathogens Inoculated to Seed Potatoes	Yield of Bulbs from One Plant (g)		Yield of Stems from One Plant (g)	
		Fresh Mass (g)	Dry Mass (g)	Fresh Mass (g)	Dry Mass (g)
10.03	Control	494.7 ^b^	84.6 ^bc^	53.5 ^c^	29.3 ^bc^
	*Fusarium oxysporum*	484.1 ^a^	82.4 ^a^	49.9 ^a^	27.3 ^a^
	*Pectobacterium corotovorum*	485.4 ^ab^	84.2 ^ab^	51.9 ^b^	28.5 ^b^
	*Rhizoctonia solani*	484.8 ^ab^	83.3 ^ab^	51.8 ^b^	28.4 ^b^
10.04	Control	505.9 ^d^	86.8 ^e^	59.9 ^e^	32.2 ^e^
	*Fusarium oxysporum*	484.5 ^ab^	83.3 ^ab^	53.6 ^c^	29.3 ^bc^
	*Pectobacterium corotovorum*	495.7 ^c^	85.2 ^cd^	55.4 ^d^	30.4 ^d^
	*Rhizoctonia solani*	495.4 ^c^	85.1 ^cd^	55.2 ^d^	30.3 ^d^
10.05	Control	517.3 ^e^	88.6 ^f^	67.1 ^h^	37.2 ^h^
	*Fusarium oxysporum*	494.6 ^bc^	84.9 ^bcd^	63.0 ^f^	34.8 ^f^
	*Pectobacterium corotovorum*	506.7 ^d^	86.6 ^de^	64.9 ^g^	35.9 ^g^
	*Rhizoctonia solani*	506.0 ^d^	86,5 ^de^	64.6 ^g^	36.0 ^g^
LSD_0.05_		10.0	1.8	1.5	1.0

Means marked with the same letters do not differ statistically at the significance level *p* = 0.05. LSD was calculated at the significance level of *p* = 0.05.

**Table 5:** Potato pathogens used in the study.

Strains	Origin
*Alternaria alternata* ŁOCK 408	Collection of Pure Cultures of Industrial Microorganisms ŁOCK at the Lodz University of Technology (Łódź, Poland)
*Fusarium oxysporum* Z154	Plant Breeding and Acclimatization Institute (IHAR)—National Research Institute (Radzików, Poland)
*Alternaria solani* Z184	
*Pectobacterium carotovorum* PCM 2056 *	Polish Collection of Microorganisms of the Hirszfeld Institute of Immunology and Experimental Therapy of the Polish Academy of Sciences (Wrocław, Poland)
*Alternaria tenuissima* DSM 63360	German Collection of Microorganisms and Cell Cultures GmbH (DSMZ, Braunschweig, Germany)
*Fusarium sambucinum* DSM 62397	
*Rhizoctonia solani* DSM 22843	
*Colletotrichum coccodes* DSM 62126	
*Phoma exigua* DSM 62040	
*Streptomyces scabiei* DSM 4077 *	

* bacteria.

## Data Availability

Not applicable.

## Supplementary Materials

The following supporting information can be downloaded at: https://www.mdpi.com/article/10.3390/molecules27123708/s1, Table S1: Volatile compounds identified from potato tubers contaminated with different phytopathogens (the results were expressed as means of peak area %, *n* = 2).
