# Supplementary material for: Volatile Organic Compounds and Physiological Parameters as Markers of Potato (Solanum tuberosum L.) Infection with Phytopathogens
Source: Molecules. 2022 Jun 9;27(12):3708. doi: 10.3390/molecules27123708 (PMC9230024; doi:10.3390/molecules27123708)
Supplement: Supplementary file 1 [file molecules-27-03708-s001.zip › molecules-1762933-supplementary.pdf]

Table S1. Volatile compounds identified from potato tubers contaminated with different phytopathogens (the results were expressed as means of peak area%, n=2)

No.	Compound name	RT	RI	Relative content (% peak area)											
				Control_0[×]	After 14-day storage										
					Control	Aa	As	At	Cc	Fo	Fs	Pc	Pe	Rs	Ss
1	β-Pinene	18.59	962	0.92	nd	0.14	nd	nd	nd	nd	0.15	0.98	0.38	nd	nd
2	(+)-epi-Bicyclosesquiphellandrene	28.47	1428	nd	nd	nd	nd	nd	nd	nd	2.60	nd	nd	nd	nd
3	1-(1-Methoxypropan-2-yloxy)propan-2-yl acetate	24.28	1159	1.58	nd	nd	nd	nd	nd	nd	1.12	nd	nd	nd	nd
4	1,1,3-Trimethylcyclopentane	4.94	712	nd	0.16	nd	nd	nd	nd	0.50	nd	nd	nd	nd	nd
5	1,2,3-Trimethylbenzene	20.23	998	nd	nd	nd	nd	15.62	nd	nd	nd	nd	nd	nd	1.00
6	1,2,3-Trimethylcyclopentane	5.90	723	nd	nd	nd	nd	nd	nd	0.45	nd	nd	nd	nd	nd
7	1,2,4-Trimethylbenzene	19.17	974	nd	nd	nd	nd	nd	14.09	nd	nd	nd	nd	29.12	nd
8	1,2,4-Trimethylcyclopentane	5.59	718	nd	nd	nd	nd	nd	nd	0.46	nd	nd	0.84	nd	nd
9	1,4-diethylbenzene	21.29	1035	nd	nd	nd	2.39	nd	nd	nd	2.08	nd	nd	nd	0.57
10	1-Butanol	3.31	633	0.52	4.53	nd	nd	nd	nd	0.58	nd	nd	nd	nd	nd
11	1-Ethyl-3,5-dimethylbenzene	21.46	1041	nd	nd	nd	nd	0.61	nd	2.34	nd	nd	nd	nd	nd
12	1-Ethyl-4-methylbenzene	18.45	958	nd	nd	nd	nd	nd	nd	1.78	nd	nd	nd	nd	nd
13	1-Methyl-3-propylbenzene	21.37	1037	nd	nd	nd	1.07	1.64	0.82	1.76	1.12	1.45	0.38	0.87	0.54
14	1-Methyl-4-propylbenzene	21.18	1031	nd	nd	nd	nd	nd	nd	nd	0.35	nd	nd	nd	nd
15	1-Methylnaphthalene	26.40	1282	nd	nd	nd	nd	nd	nd	nd	0.34	nd	nd	nd	nd
16	1-Nonanol	23.49	1119	1.49	nd	nd	nd	nd	nd	nd	nd	nd	nd	nd	nd
17	1-Octen-3-ol	18.81	966	12.20	nd	nd	nd	4.18	nd	nd	0.97	1.48	1.00	nd	nd
18	1-Octen-3-one	18.45	958	0.88	nd	1.25	10.46	nd	3.30	1.23	nd	nd	2.16	nd	nd
19	1-Octene	8.34	778	0.30	nd	nd	nd	nd	nd	nd	nd	nd	nd	nd	nd
20	1-Pentanol	6.70	744	0.52	nd	nd	nd	nd	nd	nd	nd	nd	nd	nd	nd
21	2 - Ethyl - 1,4 - dimethyl - benzene	22.07	1063	nd	nd	nd	nd	1.80	nd	nd	nd	nd	1.32	nd	nd
22	2-(Hexyloxy)ethanol	22.83	1090	0.39	nd	nd	nd	nd	nd	nd	nd	nd	nd	nd	nd
23	2,4-Dimethylheptane	11.00	825	0.40	0.23	nd	nd	nd	nd	nd	0.29	nd	2.62	1.61	0.93
24	2,4-Dimethylpentane	2.82	606	nd	nd	nd	nd	1.15	nd	0.86	nd	nd	nd	nd	nd
25	2,5-Dimethylhexane	5.38	714	nd	nd	nd	nd	nd	nd	0.51	nd	nd	nd	nd	nd
26	2,6,11-Trimethyldodecane	22.13	1065	nd	nd	nd	nd	2.61	nd	nd	nd	nd	nd	nd	nd
27	2,6-Dimethylundecane	25.34	1216	nd	0.74	nd	nd	nd	nd	nd	nd	nd	nd	nd	nd
28	2-Butanol	2.40	561	0.14	nd	nd	nd	nd	nd	nd	0.27	0.95	nd	nd	nd
29	2-Butanone	2.26	543	1.10	0.55	nd	nd	nd	nd	nd	nd	nd	nd	nd	nd
30	2-Butoxyethanol	15.19	890	nd	5.73	nd	nd	0.47	nd	0.24	nd	18.00	nd	nd	nd
31	2-Ethyl-3-hydroxyhexyl 2-methylpropanoate	27.44	1353	5.49	nd	nd	nd	nd	nd	nd	nd	nd	nd	nd	nd
32	2-Heptanone	14.20	875	0.58	nd	nd	nd	nd	nd	nd	nd	nd	nd	nd	nd
33	2-Hexanone	7.36	758	0.37	nd	nd	nd	nd	nd	nd	nd	nd	0.60	nd	nd

nd -not detected; [×] - control sample before storage

Table S1. Continued

No.	Compound name	RT	RI	Relative content (% peak area)											
				Control_0^×^	After 14-day storage										
					Control	Aa	As	At	Cc	Fo	Fs	Pc	Pe	Rs	Ss
34	2-Methyl-1-butanol	5.32	716	0.31	0.13	0.50	nd	nd	nd	nd	0.08	0.49	nd	nd	nd
35	2-Methylheptane	6.97	733	nd	nd	nd	nd	nd	nd	2.98	nd	nd	nd	nd	nd
36	2-Methylhexane	3.48	642	nd	1.07	nd	nd	2.42	0.63	4.28	nd	1.13	nd	nd	nd
37	2-Methylpropanal	1.98	507	0.33	nd	nd	nd	nd	nd	nd	nd	nd	nd	nd	nd
38	2-Methylpropanol	2.73	601	0.32	nd	nd	nd	nd	nd	nd	nd	nd	nd	nd	nd
39	2-Nonen-1-ol	21.92	1057	1.47	nd	nd	0.72	1.20	nd	nd	0.08	nd	nd	nd	nd
40	2-Octenal	21.29	1035	0.79	nd	nd	nd	nd	nd	nd	nd	nd	nd	nd	nd
41	2-Pentanone	3.59	648	nd	0.32	nd	nd	nd	nd	nd	nd	nd	nd	nd	nd
42	2-Pentylfuran	19.37	979	0.45	nd	nd	nd	nd	nd	nd	0.61	0.82	nd	nd	nd
43	2-Phenylisopropanol	21.99	1060	0.15	nd	nd	nd	nd	nd	nd	nd	nd	nd	nd	0.57
44	2-Propanone	1.67	87	1.56	nd	nd	nd	nd	nd	nd	nd	nd	nd	nd	nd
45	3,3-Dimethylpentane	5.67	721	nd	nd	nd	nd	nd	nd	0.21	nd	nd	nd	nd	nd
46	3-Carene	20.21	997	4.07	nd	3.73	9.14	nd	6.87	nd	nd	nd	6.15	nd	4.91
47	3-Hydroxy-2,4,4-trimethylpentyl 2-methylpropanoate	27.17	1334	3.55	nd	nd	3.20	nd	0.57	nd	nd	nd	nd	nd	nd
48	3-Methyl-1-butanol	5.15	712	0.83	0.14	1.15	0.30	nd	nd	nd	nd	0.27	nd	nd	0.47
49	3-Methylbenzofuran	24.01	1146	nd	nd	nd	1.44	nd	nd	nd	nd	nd	nd	nd	nd
50	3-Methylbutanal	2.94	612	1.09	nd	nd	nd	nd	nd	nd	nd	nd	nd	nd	1.26
51	2-Methylbutanal	3.10	621	2.06	nd	nd	nd	nd	nd	nd	nd	nd	nd	nd	1.64
52	3-Methylhexane	3.68	652	0.38	1.41	1.08	nd	1.95	1.10	3.02	nd	1.03	nd	nd	nd
53	3-Methyloctane	13.83	868	nd	nd	nd	nd	nd	nd	0.40	nd	nd	nd	nd	nd
54	3-Octanol	19.64	985	0.04	nd	nd	nd	nd	nd	nd	9.44	nd	nd	nd	nd
55	3-Octanone	18.84	967	nd	nd	nd	nd	nd	4.70	nd	0.55	nd	nd	nd	nd
56	4-Ethyl-1-octyn-3-ol	24.42	1166	0.56	nd	nd	nd	nd	nd	nd	nd	nd	nd	nd	nd
57	4-Heptanone	13.19	855	nd	nd	nd	nd	nd	nd	0.51	nd	nd	nd	nd	nd
58	4-Methyloctane	13.75	861	nd	nd	nd	nd	nd	0.46	0.53	nd	nd	nd	nd	nd
59	Acenaphthene	28.79	1453	nd	nd	nd	nd	nd	nd	nd	0.79	nd	nd	nd	nd
60	Acetic acid	2.36	556	2.04	nd	9.31	16.45	1.09	7.54	1.58	1.21	1.02	10.79	19.97	11.91
61	Benzaldehyde	16.83	923	nd	2.98	nd	nd	nd	nd	nd	nd	nd	nd	nd	nd
62	Benzene	3.12	622	nd	nd	nd	1.46	1.86	nd	0.60	0.43	1.48	nd	nd	nd
63	Benzeneacetaldehyde	20.37	1002	0.94	nd	nd	nd	nd	nd	nd	nd	nd	nd	nd	nd
64	Benzothiazole	24.76	1184	nd	nd	nd	nd	0.98	nd	nd	nd	nd	nd	nd	nd
65	Benzyl ethanoate	23.75	1132	0.34	nd	nd	nd	nd	nd	nd	0.16	nd	nd	nd	nd
66	Chamigrene	28.98	1468	nd	nd	nd	nd	nd	nd	nd	3.75	nd	nd	nd	nd
67	cis-1,3-Dimethylcyclopentane	3.85	662	nd	0.18	nd	nd	nd	nd	0.38	nd	nd	nd	nd	nd

nd -not detected; [×] - control sample before storage

Table S1. Continued

No.	Compound name	RT	RI	Relative content (% peak area)											
				Control_0ˣ	After 14-day storage										
					Control	Aa	As	At	Cc	Fo	Fs	Pc	Pe	Rs	Ss
68	Cyclohexane	3.28	631	nd	nd	nd	nd	0.77	nd	0.62	nd	nd	nd	nd	nd
69	Cyclohexanone	13.29	859	nd	nd	nd	nd	nd	nd	0.90	nd	nd	nd	nd	nd
70	Cyclopentanone	6.65	747	nd	nd	nd	nd	nd	nd	nd	nd	nd	nd	nd	0.53
71	D-Carvone	25.26	1211	0.81	nd	nd	nd	nd	nd	nd	nd	nd	nd	nd	nd
72	Decanal	24.77	1184	1.37	nd	2.23	5.17	1.51	2.46	0.57	1.53	nd	3.58	7.09	9.27
73	Decane	20.29	999	0.65	4.96	0.57	nd	10.36	2.17	4.08	0.63	2.25	1.94	nd	nd
74	dibenzo-furan	29.23	1487	0.53	nd	1.37	4.08	nd	2.49	nd	2.75	0.49	nd	5.83	nd
75	Dihydrocitronellol	23.70	1130	1.76	nd	nd	nd	nd	nd	nd	nd	nd	nd	nd	nd
76	Dihydromyrcenol	22.05	1062	1.37	nd	nd	nd	nd	nd	nd	nd	nd	nd	nd	nd
77	Dimethyl disulfide	5.00	709	nd	nd	nd	nd	nd	nd	nd	0.49	nd	nd	nd	nd
78	D-Limonene	20.82	1018	nd	nd	34.19	nd	nd	20.18	nd	nd	32.72	nd	nd	25.24
79	Dodecane	25.06	1200	1.16	2.37	1.14	nd	0.58	0.99	2.42	0.43	1.66	2.00	nd	1.76
80	Ethenylbenzene	13.93	871	0.55	0.53	7.08	2.36	1.15	3.62	2.18	0.35	nd	14.01	nd	1.67
81	Ethyl acetate	2.51	575	nd	0.29	nd	nd	nd	nd	nd	nd	nd	nd	nd	nd
82	Ethyl octanoate	24.72	1182	0.12	nd	nd	nd	nd	nd	nd	nd	nd	nd	nd	nd
83	Ethylbenzene	12.34	846	0.36	2.15	0.65	1.28	1.26	0.74	nd	0.27	1.01	1.19	1.62	0.37
84	Ethylcyclobutane	13.59	865	0.49	nd	nd	nd	nd	nd	nd	nd	nd	nd	nd	nd
85	Ethylcyclopentane	5.24	714	nd	0.23	nd	nd	nd	nd	0.51	nd	nd	nd	nd	nd
86	Eucalyptol	20.75	1016	nd	nd	nd	nd	nd	nd	nd	nd	nd	nd	nd	0.89
87	Heptanal	14.65	882	0.30	nd	nd	nd	nd	nd	nd	nd	nd	nd	0.92	0.92
88	Heptane	4.31	686	0.48	3.87	2.06	1.19	1.18	2.83	5.66	nd	1.41	nd	nd	nd
89	Hexadecane	30.59	1600	0.64	nd	nd	nd	nd	nd	nd	nd	nd	nd	nd	0.35
90	Hexanal	7.66	764	3.92	0.66	nd	1.20	nd	nd	nd	0.17	nd	nd	1.92	1.63
91	Hexane	2.45	567	0.13	0.21	5.70	nd	2.49	2.52	3.11	nd	nd	11.81	nd	nd
92	Hexanoic acid	19.27	977	0.12	nd	nd	nd	nd	nd	nd	nd	nd	nd	nd	nd
93	Indane	20.55	1008	0.18	7.27	nd	2.85	5.40	0.67	0.03	2.67	3.14	1.21	0.62	1.59
94	Isoborneol	24.01	1148	0.79	nd	nd	nd	nd	nd	nd	0.15	nd	nd	nd	nd
95	Isobutylbenzene	19.86	987	nd	nd	nd	nd	2.74	nd	nd	nd	nd	nd	nd	nd
96	Isopropylbenzene	15.99	916	0.07	5.60	nd	nd	0.96	nd	1.79	1.45	1.19	nd	nd	nd
97	Isopropylocyclohexane	16.15	904	nd	nd	nd	nd	nd	nd	0.20	0.59	nd	nd	nd	nd

nd -not detected; ˣ - control sample before storage

Table S1. Continued

No	Compound name	RT	RI	Relative content (% peak area)											
				Control_0ˣ	After 14-day storage										
					Control	Aa	As	At	Cc	Fo	Fs	Pc	Pe	Rs	Ss
98	Longifolene	28.16	1405	0.23	nd	nd	nd	nd	nd	0.22	0.25	nd	nd	nd	0.98
99	L-α-Terpineol	24.49	1170	0.29	nd	nd	nd	nd	nd	nd	nd	nd	nd	nd	nd
100	m-Cymene	20.50	1006	3.11	0.49	0.90	1.65	2.00	0.95	6.12	0.54	1.12	1.75	0.83	1.03
101	Methylbenzene	6.18	733	1.98	0.71	3.52	6.74	3.96	3.09	3.19	0.79	3.47	5.56	5.39	2.64
102	Methylcyclopentane	2.76	603	nd	nd	nd	nd	0.70	nd	0.88	nd	nd	nd	nd	nd
103	Methylocyclohexane	4.83	706	0.19	2.92	1.65	0.69	4.64	2.08	8.78	nd	0.91	nd	nd	nd
104	m-Xylene	12.97	855	0.38	1.90	nd	nd	0.22	nd	1.86	nd	1.65	1.92	2.84	nd
105	Naphthalene	24.17	1154	1.81	nd	nd	nd	nd	nd	nd	2.01	nd	nd	nd	nd
106	Nonanal	22.66	1084	1.78	1.57	3.04	5.59	1.91	3.79	nd	1.31	1.95	4.57	8.62	9.98
107	Nonane	15.73	899	0.22	2.08	0.41	nd	0.18	nd	1.51	0.26	0.83	3.74	nd	nd
108	Octanal	19.50	982	0.47	nd	0.23	1.63	nd	0.69	nd	0.27	0.07	0.65	1.77	3.07
109	Octane	9.17	795	0.36	nd	nd	nd	nd	nd	2.31	nd	nd	0.97	nd	nd
110	o-Xylene	14.15	880	nd	4.83	nd	nd	3.10	nd	3.82	1.59	nd	3.59	nd	2.15
111	p-Cymene	20.42	1003	0.61	2.27	0.67	1.22	1.77	1.26	1.11	1.06	2.01	1.64	1.51	2.82
112	Pentadecane	29.38	1500	1.25	nd	0.06	nd	0.52	nd	nd	nd	0.57	nd	0.45	nd
113	Pentanal	3.73	655	0.22	nd	nd	nd	nd	nd	nd	nd	nd	nd	nd	nd
114	Pentane	1.71	89	3.74	0.58	3.77	nd	nd	nd	nd	nd	nd	nd	nd	nd
115	Pentanoic acid	14.98	887	0.53	nd	nd	nd	nd	nd	nd	nd	nd	nd	nd	nd
116	Phenol	18.85	967	nd	1.31	nd	nd	nd	nd	nd	nd	nd	nd	nd	nd
117	Propylbenzene	17.37	935	1.08	21.99	nd	5.05	7.13	nd	12.68	5.25	6.56	nd	nd	2.00
118	Propylcyclohexane	16.72	920	nd	0.89	nd	nd	nd	0.23	nd	nd	nd	nd	nd	nd
119	p-Xylene	12.91	854	0.88	6.11	2.83	5.27	3.80	2.01	4.32	1.22	3.98	3.62	4.36	2.81
120	Spiro[3.4]octan-5-one	20.69	1013	nd	nd	nd	nd	nd	nd	nd	4.42	nd	nd	nd	nd
121	Tetradecane	28.09	1400	1.15	0.23	0.17	0.36	nd	0.37	nd	0.23	0.30	nd	nd	0.58
122	trans-1,3-Dimethylcyclopentane	3.79	658	nd	0.28	nd	nd	nd	nd	0.48	nd	nd	nd	nd	nd
123	Tridecane	26.67	1300	0.57	0.60	0.38	nd	0.49	nd	0.57	0.35	nd	1.20	nd	0.65
124	Undecane	23.09	1100	1.10	4.93	nd	nd	nd	nd	nd	nd	nd	nd	nd	nd
125	Valencene	29.25	1489	nd	nd	nd	nd	1.13	nd	nd	26.53	nd	nd	nd	nd
126	α-Cubebene	27.76	1376	nd	nd	nd	0.81	nd	nd	0.09	7.76	nd	nd	1.90	nd
127	α-Guaiene	28.71	1447	nd	nd	nd	nd	nd	nd	nd	7.11	nd	nd	nd	nd
128	α-Pinene	16.85	923	13.21	nd	10.23	6.24	1.77	6.79	4.77	1.11	3.62	8.82	2.77	3.27
129	β-Cedrene	28.34	1419	nd	nd	nd	nd	0.69	nd	nd	nd	nd	nd	nd	nd
130	γ-Terpinene	21.72	1050	0.93	nd	nd	nd	nd	nd	nd	0.07	nd	nd	nd	nd

nd -not detected; ˣ - control sample before storage
